# Determinants of breast size in Asian women

**DOI:** 10.1038/s41598-018-19437-4

**Published:** 2018-01-19

**Authors:** Li Yan Lim, Peh Joo Ho, Jenny Liu, Wen Yee Chay, Min-Han Tan, Mikael Hartman, Jingmei Li

**Affiliations:** 1 Department of Surgery, Yong Loo Lin School of Medicine, National University of Singapore, Singapore, Singapore; 2 Saw Swee Hock School of Public Health, National University of Singapore, Singapore, Singapore; 3 National Cancer Centre, Singapore, Singapore; 4 Institute of Bioengineering and Nanotechnology, Singapore, Singapore; 5 Human Genetics, Genome Institute of Singapore, Singapore, Singapore

## Abstract

Breast size as a risk factor of breast cancer has been studied extensively with inconclusive results. Here we examined the associations between breast size and breast cancer risk factors in 24,353 Asian women aged 50 to 64 years old enrolled in a nationwide mammography screening project conducted between October 1994 and February 1997. Information on demographic and reproductive factors was obtained via a questionnaire. Breast size was ascertained as bust line measured at study recruitment and total breast area measured from a mammogram. The average bust line and total breast area was 91.2 cm and 102.3 cm^2^, respectively. The two breast measurements were moderately correlated (Spearman correlation coefficient = 0.65). Age, BMI, marital and working status were independently associated with bust line and total breast area. In the multivariable analyses, the most pronounced effects were observed for BMI (24.2 cm difference in bust line and 39.4 cm^2^ in breast area comparing women with BMI ≥30 kg/m^2^ to BMI <20 kg/m^2^). Ethnicity was a positive predictor for total breast area, but not bust line.

## Introduction

Breast development occurs during puberty in girls, after which the enlarged breasts are retained throughout adulthood. Breast size in adult women is associated with perceived femininity and is sexually important in many cultures^1,2^. Apart from being a potential marker of health and fertility from an anthropological point of view, breast size has been shown to affect women’s quality of life and well-being in many ways – socially, psychologically and physically^3^. For example, several studies on sport and exercise participation in adolescent girls revealed large breasts to be a cause of physical strain and a major reason why they do not take part in physical activity^4,5^. Back problems are also more common among women who are more well-endowed^6^. In terms of breast health, breast size has been studied extensively as a risk factor of breast cancer, but the results are inconclusive^7^.

Breast size is a highly heritable trait. A twin study previously estimated the heritability of bra cup size to be 56%^8^. Several genome-wide association studies have also identified common genetic variants associated with breast size^9,10^. Asian women are typically less well-endowed^11,12^ than women of Caucasian ancestry. Several demographic, reproductive and lifestyle factors have been suggested to influence breast size, but most of these links are anecdotal in nature. Few studies have examined the associations rigorously^13,14^, and fewer have been performed in Asian populations. Here, we aim to identify non-genetic determinants of breast size using a large cohort of women in Southeast Asia. In addition, we examined how the candidate predictors of breast size affects the different tissue components that make up the breast.

## Methods

### Study population

The study population was made up of Singaporean women aged 50 to 64 years old on 1 October 1994 who enrolled in the Singapore Breast Cancer Screening Programme (SBCSP). SBCSP was a prospective nationwide mammography screening project conducted between October 1994 and February 1997, which has previously been described in detail^15^. From 166,600 women identified to be eligible in a comprehensive population registry for randomization to breast screening or observation, 69,473 women were randomly chosen and offered free mammography screening. Women who consented to take part in the study were interviewed by a nurse or study coordinator and asked to complete a questionnaire regarding their demographic and anthropometric measures as well as family and reproductive history before being invited for a one-time mammogram examination. Participation rate (eligible women who took up the invitation for free mammography screening) was 41.7%.

### Exclusions

Eligible participants were first asked five questions: 1) Have you ever been diagnosed with breast cancer? 2) Have you ever been diagnosed with other cancer (except non-melanoma skin cancers)? 3) Have you had a mammogram done in the past one year? 4) Have you had a breast biopsy in the past one year? 5) Are you likely to be pregnant? Women were excluded if they answered “Yes” to any of the five questions. Of 28,672 women who returned questionnaire data, mammographic density measurements were available for 24,363 participants (85.0%). Mammograms were either unavailable, or the image quality of the scanned mammogram was too poor for the accurate assessment of mammographic density for 4,309 women. Ten additional women were excluded due to missing information on bust line, weight or height. The final analytical dataset consisted of 24,353 participants.

### Assessment of breast size

**Figure 1:**
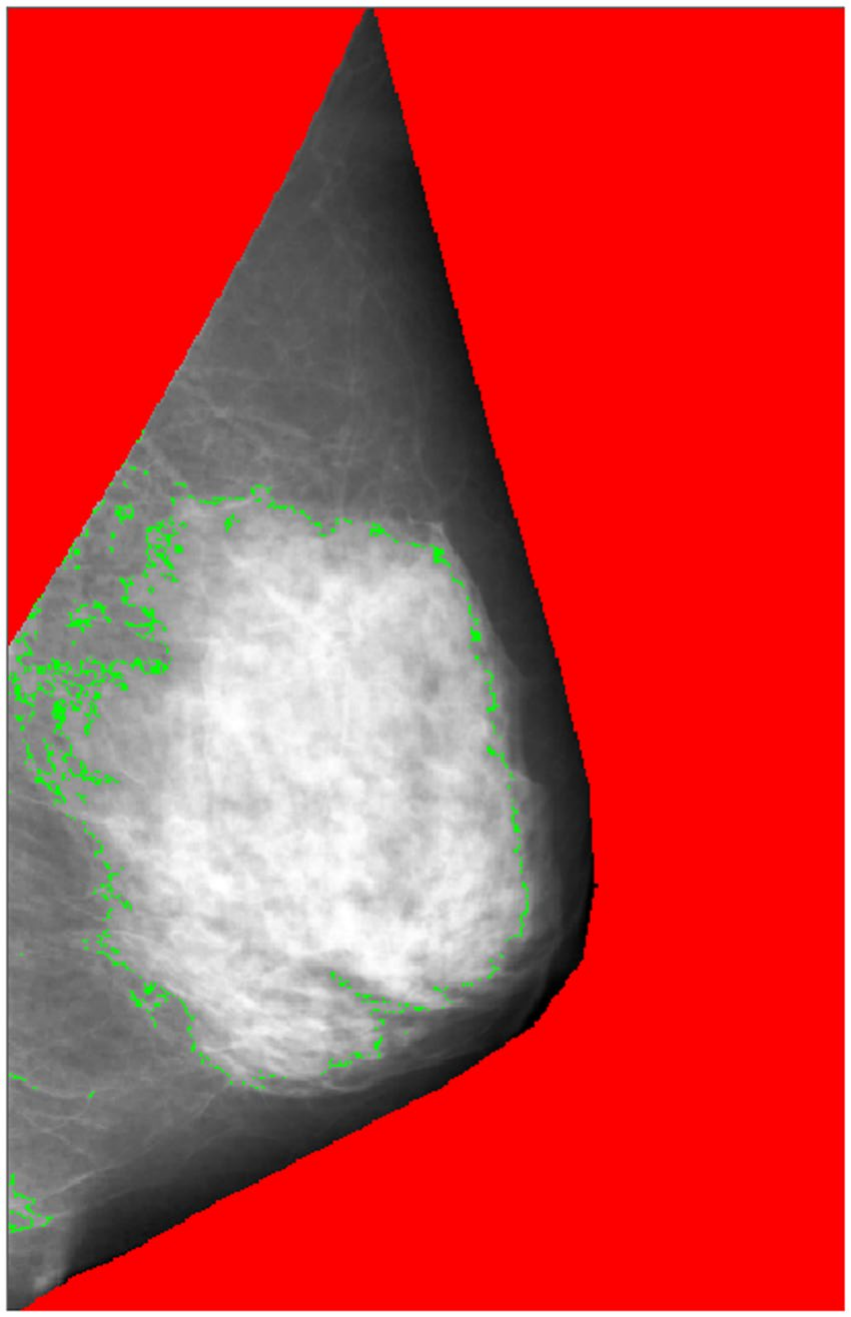
Breast area, dense area (appears white) and nondense area (appears dark) on a mammogram.

Bust line is measured as the chest circumference of the fullest part of a woman’s breasts at study entry. The mean of two measurements of the bust line were taken. Total breast area, dense area and nondense area were measured from mammograms taken from the medio-lateral oblique view (Fig. 1). Dense area (radiodense glandular tissue) and nondense area (radiolucent fat) are separate components of the total breast area captured on a mammogram. Details on the assessment of the mammographic total breast area and dense and nondense components have been described previously^16^. Briefly, original film mammograms from SBCSP were retrieved and digitized between February 2012 through February 2013 using the 2905 Laser Film Digitizer (Array Corporation, Model 2905, Tokyo, Japan), with a sampling pitch of 50 micrometers and a gray-scale contrast resolution of 12 bits. Total mammographic breast area and dense area were then estimated using a fully-automated thresholding method described by Li *et al*.^17^ for mammograms from each breast before mean values were computed. Mean nondense area was derived by taking the difference between mean total breast area and mean dense area. The concordance correlation coefficients for total and absolute dense breast area measured by Li *et al*.’s method and the gold standard method (Cumulus) were 0.95 (95% CI: 0.94–0.95) and 0.85 (95% CI: 0.83–0.87), respectively.

### Assessment of potential determinants of breast size

Information on self-reported demographic (age [<55, 55–59, ≥ 60], ethnicity [Chinese, Indian, Malay, Others], marital status [married, divorced, single], occupation [housewife, employed, unemployed, retired]), reproductive and hormonal factors (number of children [“How many deliveries (of at least 6.5 months or 28 weeks of gestation) have you had?”], ever breastfeeding [“Did you breast feed any of your children?”, yes/no], oral contraceptive use [“Did you ever use oral pill as a form of contraceptive?”, yes/no], ever hormone replacement therapy use [“Have you ever been on hormone replacement therapy?”, yes/no]) were obtained from the SBCSP questionnaire at the time of breast size ascertainment (mammography). Participants were also asked about family history of breast cancer (“Has your mother/any of your sisters/any of your daughters ever had breast cancer?”, yes/no) in the questionnaire. Body mass index (kg/m^2^) was computed based on self-reported measures of height and weight.

### Statistical analysis

Spearman correlation coefficients from non-parametric rank-based tests between bust line, total breast area, nondense area and dense area were calculated and correlations were visualized using scatter diagrams. To correct for non-normality, total breast area and dense area were square-root transformed (Supplementary Figure 1). Associations between various factors described above and different measurements of breast size (bust line, total breast area, dense area and nondense area) were first examined using simple linear regression models without any adjustments. Coefficients were back-transformed using the equation, 2***ab*** + ***b***^2^, where ***a*** is the intercept *and* ***b*** is the coefficient itself. Categories of each factor are mutually exclusive, hence back transformation rely on the intercept and the coefficient itself. The associations were also examined using multivariable linear regression models adjusting for all factors found to be significantly associated with any measure of breast size in the unadjusted analyses. To facilitate comparison of the results between different breast size phenotypes, log-modulus transformation was applied to the effect sizes (Z scores) from the multivariable adjusted analyses. The transformation takes the logarithm of the absolute value plus 1. If the original value was negative, the sign was preserved by multiplying the transformed value by −1. All of the statistical tests were 2-sided, and the differences were considered statistically significant at a P-value of <0.05.

This study was approved by the Institutional Review Board at the National University of Singapore. No informed consent was required as the data were analysed anonymously. All analyses were performed in accordance with the relevant guidelines and regulations.

### Data availability

The datasets used and/or analysed during the current study are available from the corresponding author on reasonable request.

## Results

### Singapore Breast Cancer Screening Programme (SBCSP)

The characteristics of the 24,353 SBCSP participants included in our study are described in Table 1. Chinese form the largest ethnic group, comprising 85.1% of the population included in our analyses. Malays, Indians and other ethnic groups each made up 5% (~1,000 women) of the study population. Four in five women were married. A third of the women were in the work force while over 60% of the women reported being housewives. Seventy percent of the women had at least three children. Of the women who had at least one child (*n* = 22, 417), two-thirds reported ever breastfeeding. Six in ten women had never taken oral contraceptives. The ever use of hormone replacement therapy (HRT) was low at 13.6% (*n* = 3, 301). Less than 3% of the women reported that a first-degree relative have had a breast cancer. Women in the SBSCP study had, on average, a mean bust line of 91.2 cm (standard deviation [SD] = 8.1) at study entry. The average difference between two bust line measurements used to compute the mean bust line was −0.047 (0.44) cm. The mean total breast area, nondense area and dense area captured on mammograms were 102.3 (20.8), 81.3 (24.3) and 21.0 (13.2) cm^2^, respectively.

**Table 1:** Description of the Singapore Breast Cancer Screening Project (SBCSP) study (*n* = 24,353) and the associations with different breast size (bust line in cm or total breast area on mammogram in cm^2^) or breast component (dense area or nondense area in cm^2^) measurements from unadjusted linear regression models. Beta and standard errors for breast area and dense area correspond to analyses performed on the square-root transformed scale. *Denotes back-transformed values for ease of interpretation.

	*n* (%)	Bust line		Breast area			Nondense		Dense area		
		Beta (SE)	p-value	Beta*	Beta (SE)	p-value	Beta (SE)	p-value	Beta*	Beta (SE)	p-value
**Age at entry**											
<55	7377 (30.3)	Referent			Referent		Referent			Referent	
55–59	9034 (37.1)	0.29 (0.13)	0.022	2.61	0.13 (0.02)	<0.001	7.32 (0.37)	<0.001	−4.61	−0.50 (0.02)	<0.001
≥60	7942 (32.6)	1.13 (0.13)	<0.001	6.03	0.30 (0.02)	<0.001	15.41 (0.38)	<0.001	−9.23	−1.06 (0.02)	<0.001
P-trend		<0.001			<0.001		<0.001			<0.001	
**Ethnicity**											
Chinese	20719 (85.1)	Referent			Referent		Referent			Referent	
Malay	1313 (5.4)	3.70 (0.23)	<0.001	18.82	0.91 (0.03)	<0.001	24.30 (0.66)	<0.001	−5.23	−0.64 (0.04)	<0.001
Indian	1132 (4.6)	2.63 (0.25)	<0.001	18.56	0.89 (0.03)	<0.001	19.59 (0.70)	<0.001	−0.78	−0.09 (0.04)	0.033
Other	1189 (4.9)	3.07 (0.24)	<0.001	18.04	0.87 (0.03)	<0.001	20.63 (0.69)	<0.001	−2.51	−0.29 (0.04)	<0.001
**Marital status**											
Married	19484 (80.0)	Referent			Referent		Referent			Referent	
Divorced	3807 (15.6)	−4.07 (0.26)	<0.001	−7.62	−0.39 (0.03)	<0.001	−15.08 (0.76)	<0.001	7.79	0.81 (0.04)	<0.001
Single	1062 (4.4)	0.54 (0.14)	<0.001	2.50	0.12 (0.02)	<0.001	5.08 (0.43)	<0.001	−2.52	−0.30 (0.02)	<0.001
**Occupation**											
Housewife	15379 (63.2)	Referent			Referent		Referent			Referent	
Employed	7918 (32.5)	−1.66 (0.11)	<0.001	−5.49	−0.27 (0.01)	<0.001	−9.65 (0.33)	<0.001	4.05	0.46 (0.02)	<0.001
Unemployed	91 (0.4)	−3.42 (0.85)	<0.001	−4.92	−0.24 (0.10)	0.019	−10.12 (2.51)	<0.001	4.94	0.55 (0.14)	<0.001
Retired	965 (3.9)	−2.91 (0.27)	<0.001	−4.87	−0.24 (0.03)	<0.001	−9.04 (0.79)	<0.001	4.07	0.46 (0.05)	<0.001
**Body mass index**											
≥30	2151 (8.8)	7.91 (0.13)	<0.001	16.96	0.78 (0.02)	<0.001	19.25 (0.49)	<0.001	−1.96	−0.24 (0.03)	<0.001
25–29	8158 (33.5)	Referent			Referent		Referent			Referent	
20–24	11425 (46.9)	−7.52 (0.07)	<0.001	−13.78	−0.68 (0.01)	<0.001	−16.55 (0.29)	<0.001	2.61	0.30 (0.02)	<0.001
<20	2619 (10.8)	−16.35 (0.12)	<0.001	−27.53	−1.41 (0.02)	<0.001	−33.91 (0.45)	<0.001	6.36	0.70 (0.03)	<0.001
P-trend		<0.001			<0.001		<0.001			<0.001	
**Number of children**											
0	1936 (7.9)	Referent			Referent		Referent			Referent	
1	1344 (5.5)	0.99 (0.29)	<0.001	2.61	0.13 (0.04)	<0.001	5.00 (0.84)	<0.001	−2.53	−0.25 (0.05)	<0.001
2	4011 (16.5)	1.28 (0.22)	<0.001	2.00	0.10 (0.03)	<0.001	5.52 (0.66)	<0.001	−3.83	−0.38 (0.04)	<0.001
≥3	17062 (70.1)	3.61 (0.19)	<0.001	6.08	0.30 (0.02)	<0.001	15.88 (0.57)	<0.001	−10.05	−1.08 (0.03)	<0.001
P-trend		<0.001			<0.001		<0.001			<0.001	
**Age at first birth (in subset of women with children)**											
<22	8296 (37)	Referent			Referent		Referent			Referent	
22–26	8018 (35.8)	−1.99 (0.12)	<0.001	−6.24	−0.31 (0.02)	<0.001	−9.88 (0.36)	<0.001	3.38	0.41 (0.02)	<0.001
≥27	6103 (27.2)	−3.32 (0.13)	<0.001	−9.15	−0.45 (0.02)	<0.001	−15.23 (0.39)	<0.001	5.95	0.69 (0.02)	<0.001
Ptrend		<0.001			<0.001		<0.001			<0.001	
**Breastfeed (in subset of women with children)**											
Yes	15459 (63.5)	Referent			Referent		Referent			Referent	
No	6958 (28.6)	−1.75 (0.12)	<0.001	−4.69	−0.23 (0.01)	<0.001	−8.13 (0.34)	<0.001	3.37	0.39 (0.02)	<0.001
**Menopausal status**											
Pre-menopausal	2574 (10.6)	Referent			Referent		Referent			Referent	
Post-menopausal	21779 (89.4)	0.30 (0.17)	0.076	4.22	0.21 (0.02)	<0.001	13.41 (0.50)	<0.001	−9.14	−0.96 (0.03)	<0.001
**Oral contraceptives**											
No	15059 (61.8)	Referent			Referent		Referent			Referent	
Yes	9294 (38.2)	0.46 (0.11)	<0.001	−0.44	−0.02 (0.01)	0.094	−0.05 (0.32)	0.869	−0.42	−0.05 (0.02)	0.009
**Hormone replacement therapy**											
No	21052 (86.4)	Referent			Referent		Referent			Referent	
Yes	3301 (13.6)	−1.73 (0.15)	<0.001	−4.41	−0.22 (0.02)	<0.001	−9.99 (0.45)	<0.001	5.54	0.60 (0.03)	<0.001
**Family history of breast cancer**											
No	23729 (97.4)	Referent			Referent		Referent			Referent	
Yes	624 (2.6)	−0.74 (0.33)	0.025	−0.72	−0.04 (0.04)	0.374	−4.45 (0.99)	<0.001	3.91	0.43 (0.06)	<0.001

### Correlations between different breast size measurements

**Figure 2:**
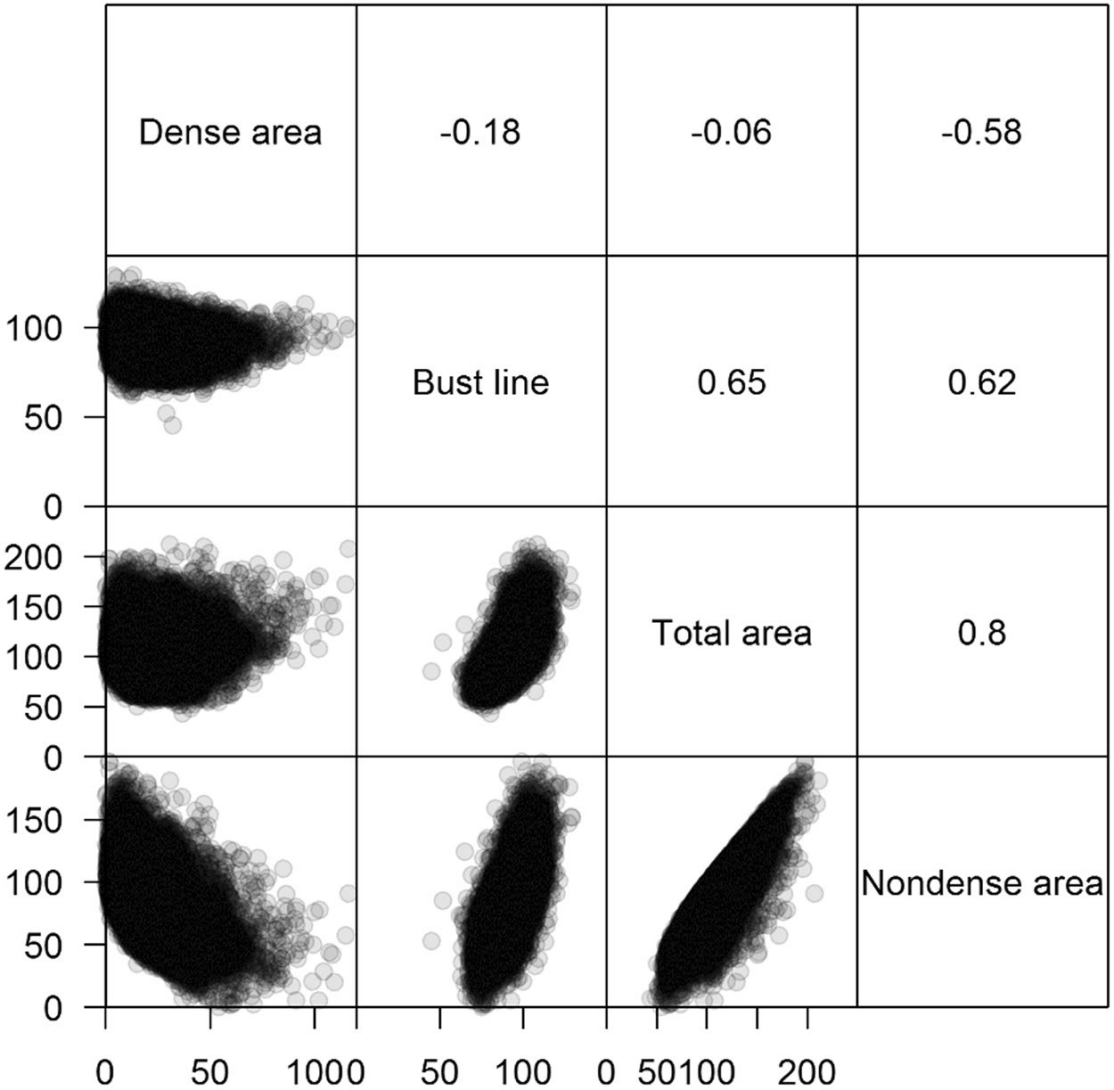
Correlations between different breast size measurements (bust line, total breast area, nondense area and dense area). Spearman correlations are presented in the upper triangle. Scatter diagrams are plotted in the lower triangle.

The highest correlation was found between total breast area and nondense area (Spearman correlation coefficient = 0.80), followed by total breast area and bust line (Spearman correlation coefficient = 0.65) (Fig. 2). Nondense area explained 70.5% of the variance of total breast area (R-squared from linear regression model). Dense area was negatively correlated with nondense area (Spearman correlation coefficient = −0.58). There was little correlation between either total breast area or bust line with dense area.

### BMI is a predictor of breast size

The directions and ranking of results from univariate association analyses between different covariates studied and bust line, total breast area and nondense area were in agreement (Table 1). On the contrary, the directions were flipped for all associations with dense area (Table 1). Older age, being non-Chinese, being married, being a housewife, high BMI, having more children, ever breastfeeding, and no HRT use were separately associated with increased bust line, total breast area and nondense area. Being postmenopausal was significantly associated with higher total breast area and lower nondense area (Table 1). In the unadjusted analyses, the most pronounced effects were observed for BMI (~24 cm difference in bust line, 44.5 cm^2^ in total breast area and 53.1 cm^2^ in nondense area between women with BMI ≥30 kg/m^2^ compared to women with BMI <20 kg/m^2^), ethnicity (~3 cm difference in bust line, ~18 cm^2^ in total breast area and ~20 cm^2^ in nondense area between non-Chinese and Chinese women), and number of children (3.6 cm difference in bust line, 6.1 cm^2^ in total breast area and 15.9 cm^2^ in nondense area for women with at least 3 children compared to women with no children) (Table 1). In a subset of 21,073 women with children, older age at first birth and never breastfed were associated with smaller bust line, smaller total breast area, smaller total nondense area and bigger dense area (Table 1).

**Figure 3:**
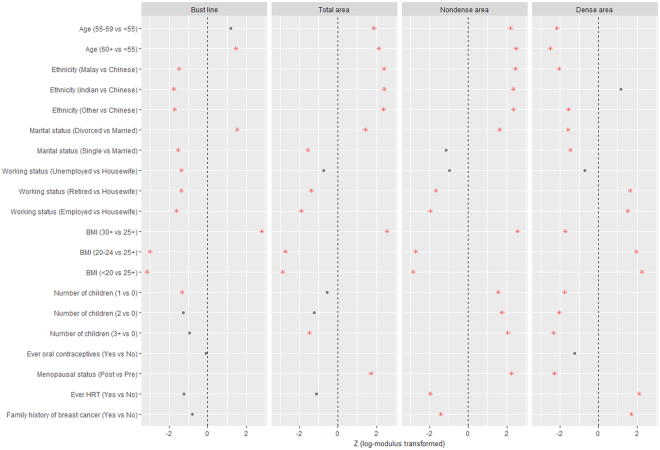
Determinants of breast size and breast components – log-modulus transformed results (Z scores) from multivariable adjusted analyses (*n* = 24,353). Red asterisks denote significant independent associations (P < 0.05). See Supplementary Table 1 for regression estimates of breast size and breast component measurements.

**Figure 4:**
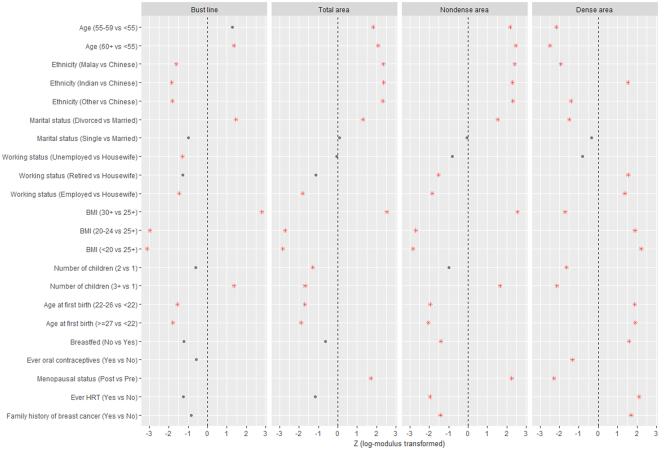
Determinants of breast size and breast components – log-modulus transformed results (Z scores) from multivariable adjusted analyses in a subset of women with children (*n* = 21,073). Red asterisks denote significant independent associations (P < 0.05). See Supplementary Table 2 for regression estimates of breast size and breast component measurements.

In the multivariable adjusted results (Fig. 3 and Supplementary Table 1), the variables found to remain significantly associated with bust line, total breast area and nondense area included marital status (divorced vs married), working status (retired vs housewife and employed vs housewife) and BMI. After adjustments, the most pronounced effects were observed for BMI (24.2 cm difference in bust line and 39.4 cm^2^ in breast area comparing women with BMI ≥30 kg/m^2^ to BMI <20 kg/m^2^). Age ethnicity and menopausal status remained positively associated with total breast area and nondense area with similar effect sizes (Z scores), but not bust line. Number of children was not independently associated with bust line, although having three or more children significantly decreased total breast area. In a subset of 21,073 women with children, older age at first birth remained significantly associated with smaller bust line, smaller total breast area, smaller total nondense area and bigger dense area in the multivariable model (Fig. 4 and Supplementary Table 2). However, ever breastfeeding was no longer significantly associated with either bust line or total breast area (Fig. 4 and Supplementary Table 2).

### Women with more children or a family history of breast cancer were associated with decreased dense breast area and increased nondense breast area

The effect sizes of the associations between different covariates and dense and nondense area were in opposite directions (Fig. 3 and Supplementary Table 1). While number of children was not strongly associated with either bust line or total breast area, women with more children had on average significantly higher nondense area and lower dense area. Similarly, although a family history of breast cancer was not significantly associated with either bust line or total breast area, significant associations with nondense (inverse association) and dense area (positive association) were found.

## Discussion

Different measurements of breast size, in particular, bust line and total breast area, were only moderately correlated (Spearman correlation coefficient = 0.65). In this large cohort of Singaporean women invited for screening mammography, fatty tissue reflected as nondense area constituted a large proportion of the total breast area captured on mammograms. Variables found to remain significantly associated with bust line, total breast area, and nondense area included BMI, marital status, and working status. Age, ethnicity, and number of children were significant predictors of breast area and nondense area, but not bust line.

Breast size ascertainment is not an exact science. Previous studies have used self-reported breast size, bra cup size, chest circumference and breast size measured from mammograms as approximations of breast size^7^. The main advantage of determining breast size from mammograms over other methods is the objectivity and a common unit of measurement (cm^2^). “Vanity sizing”, a phenomenon where different bra manufacturers follow different measurements for identically labelled bra sizes, makes it difficult to generalize study-specific findings to other populations^18^. Bust line, which uses a formal unit of measurement (cm), is arguably more objective than bra cup or band size, but is subjected to confounding by chest circumference below the bust line. Given the low correlation between bust line and total breast area found in our study, it is therefore difficult to compare between studies that look at different measures of breast size. Nonetheless, the general relationship between factors and breast size remains similar.

This study, which comprises women aged 50 to 64 years old recruited from 1994 to 1997, is made up of an Asian population of ethnic Chinese, Malay, Indian and Eurasian women with distinct demographic differences from Western screening mammography populations that have previously been studied. For example, 80% of women in this study were married, a much larger proportion than the ~65% marriage rate observed for predominantly White screening mammography populations in North America^19^. Marriage rates in European screening mammography populations vary, ranging between 50–75%^20,21^. Seventy percent of the women in this study (50 to 64 year old) had at least three children, while the proportion was less than a third of the attendees of screening mammography in Sweden between 1988 to 1997^21^. Only 32.5% of the Singaporean women in this study were gainfully employed, compared to ~80% of the women described in the Swedish screening mammography study^21^. Prevalence of HRT use is also known to be lower in Asian women compared to Caucasian women^22^.

Our findings on determinants of breast size in Singaporean women agree with what has been published in literature in Caucasian populations^8,14^. Firstly, the strongest predictor of breast size was BMI, which has been shown previously to share a significant genetic component with breast size in Caucasian women^8^. Secondly, according to a European population-based study conducted on women attending breast screening, one in five women experienced an increase in breast size after menopause^14^, which corroborates our finding on the association between higher age and larger breast area.

It is commonly known that childbearing can change breast size and appearance^23–25^. Breast enlargement and loss of firmness are the most common changes experienced by women post-pregnancy^25^. A German study estimated an increase of ~100cc regardless of the initial breast volume before pregnancy^23^. Breast size and stiffness changes were not associated with breastfeeding^24–26^. To our knowledge, the effect of pregnancy and lactation on breast size many years after childbirth has not been examined. In our study, while number of children was not an independent predictor of bust line after adjusting for other factors, having three or more children was significantly associated with smaller total breast area. In addition, older age at first birth among women with children was associated with reduced breast size. This finding is in contrast to the enlargement effect observed directly after pregnancy by other studies^25^. Future studies investigating longitudinal changes in breast size over a woman’s life time will be needed to clarify these conflicting results.

Total mammographic breast area is made up of dense and nondense components, which are known to be associated with breast cancer risk in different directions^27^. Dense tissue is associated with an increase in breast cancer risk, while nondense tissue is associated with a decrease in breast cancer risk^28^. It is likely that the hypothesized association between breast size and breast cancer risk pursued by many studies^7^ is an interplay between age, BMI, dense and nondense components of the breast. While ethnic variations in percent mammographic density, absolute dense and nondense area have been reported for a comparable Southeast Asian study^29^, breast size differences among ethnic groups have not been reported. However, Mariapun *et al*. observed that adjusted nondense area (which was highly correlated with total breast area in our study) was significantly lower in Chinese women compared to their Malay and Indian counterparts^29^. In a study of ethnic differences in percentage body fat (which influences proportion of nondense tissue) in Singapore, Deurenberg-Yap showed that for the same age, gender and BMI, Indians have a higher percentage body fat (and hence larger proportion of nondense tissue) than Malays, who in turn have a slightly higher percentage body fat than Chinese^30^. After controlling for age and gender, Chinese, Malays and Indians were all shown to have considerably higher percentage body fat for the same BMI than Caucasians^30^, which is consistent with the corresponding breast cancer risks observed for different ethnic groups in Singapore and Asians in general compared to Caucasians^31^.

A strength of the study is the size of the study population. Heng *et al*. have previously published a study on the determinants of mammographic features (percent mammographic density, absolute dense area and absolute nondense area) in a subset of 803 Chinese women in the SBCSP study^32^. We have then expanded the study by retrieving, digitizing and measuring mammograms for more than 24,000 participants, and is now one of the largest Asian study on the determinants of breast size and breast composition. We compared two different measures of breast size in this study (bust line and breast area on mammogram) and also examined the proportions of dense and nondense components.

While it is of merit to derive objective breast size measurements from mammograms as a large number of women will invariably get a mammogram taken for screening or diagnostic purposes, a resulting limitation is that the study population will be largely restricted to older women who are of mammography screening age. The use of a mediolateral oblique (angled) view, instead of a cranial-caudal (from above) view, may also impact the results. However, mammographic density measurements obtained from the two different views have been shown to be highly correlated (r > 0.8)^33^. In addition, the mammograms used in this study were collected before the adoption of digital mammography and development of methods to measure volumetric breast density. Hence, volumetric breast size, which has the advantage of taking into account breast thickness (i.e. variation due to different levels of compression), was not examined^34^. Information on previous breast augmentation or reduction surgery was not collected.

Although measurements of breast size, total breast area and bust line were only moderately correlated, the determinants of breast size found for Singaporean women were similar to those observed for Western populations. Local ethnic differences in breast size, measured as total breast area on a mammogram, persisted even after adjusting for BMI and other factors.

## Electronic supplementary material

Supplementary File
