# Supplementary material for: Determinants of breast size in Asian women
Source: Sci Rep. 2018 Jan 19;8:1201. doi: 10.1038/s41598-018-19437-4 (PMC5775321; doi:10.1038/s41598-018-19437-4)
Supplement: Supplementary file 1 — Supplementary File [file 41598_2018_19437_MOESM1_ESM.docx]

**Determinants of breast size in Asian women**

Li Yan Lim^1#^ li_yan_lim@nuhs.edu.sg

Peh Joo Ho^2#^ pehjoo_ho@u.nus.edu

Jenny Liu^2^ jenny_liu@nus.edu.sg

Wen Yee Chay^3^ chay.w.y@nccs.com.sg

Min-Han Tan^3,4^ mhtan@ibn.a-star.edu.sg

Mikael Hartman^1,2^ ephbamh@nus.edu.sg

Jingmei Li^1,5^ * lijm1@gis.a-star.edu.sg

# Equal contribution.

^1^ Department of Surgery, Yong Loo Lin School of Medicine, National University of Singapore, Singapore

^2^ Saw Swee Hock School of Public Health, National University of Singapore, Singapore

^3^ National Cancer Centre, Singapore, Singapore

^4^ Institute of Bioengineering and Nanotechnology, Singapore

^5^ Human Genetics, Genome Institute of Singapore, Singapore

*Correspondence to Dr Jingmei Li, Genome Institute of Singapore, 60 Biopolis Street, Genome, #02-01, Singapore 138672, Singapore, Tel: +65 6808 8312, Fax: +65 6808 8306. Email: lijm1@gis.a-star.edu.sg

**Supplementary Figure 1.** Distributions of different breast size and breast component measurements.


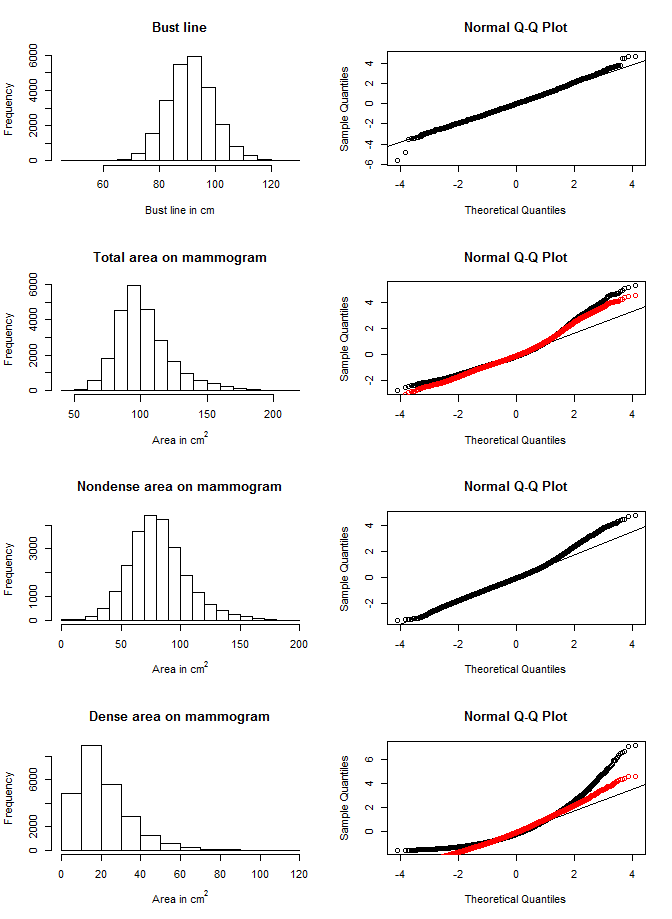


**Supplementary Table 1**. Multivariable adjusted linear regression estimates of breast size and breast component measurements (*n*=24,353). Breast size and dense area were square-root transformed. * denotes back-transformed values for ease of interpretation.

|  | **Bust line (cm)** | | | **Breast area (cm^2^)** | | | | **Nondense area (cm^2^)** | | | **Dense area (cm^2^)** | | | |
| --- | --- | --- | --- | --- | --- | --- | --- | --- | --- | --- | --- | --- | --- | --- |
|  | **BETA** | **SE** | **P** | **BETA*** | **BETA** | **SE** | **P** | **BETA** | **SE** | **P** | **BETA*** | **BETA** | **SE** | **P** |
| **Age (55-59 vs <55)** | 0.13 | 0.08 | 0.113 | 1.96 | 0.10 | 0.01 | <0.001 | 4.75 | 0.31 | <0.001 | -3.78 | -0.30 | 0.02 | <0.001 |
| **Age (60+ vs <55)** | 0.26 | 0.09 | 0.003 | 3.92 | 0.19 | 0.01 | <0.001 | 10.16 | 0.34 | <0.001 | -8.86 | -0.74 | 0.02 | <0.001 |
| **BMI (30+ vs 25-29)** | 7.98 | 0.13 | <0.001 | 14.15 | 0.67 | 0.02 | <0.001 | 16.29 | 0.45 | <0.001 | -2.04 | -0.16 | 0.03 | <0.001 |
| **BMI (20-24 vs 25-29)** | -7.48 | 0.08 | <0.001 | -12.15 | -0.62 | 0.01 | <0.001 | -14.00 | 0.27 | <0.001 | 2.17 | 0.17 | 0.02 | <0.001 |
| **BMI (<20 vs 25-29)** | -16.26 | 0.12 | <0.001 | -25.29 | -1.33 | 0.02 | <0.001 | -30.54 | 0.42 | <0.001 | 6.78 | 0.51 | 0.03 | <0.001 |
| **Ethnicity (Malay vs Chinese)** | -0.46 | 0.15 | 0.002 | 11.60 | 0.55 | 0.02 | <0.001 | 14.81 | 0.53 | <0.001 | -4.79 | -0.39 | 0.04 | <0.001 |
| **Ethnicity (Indian vs Chinese)** | -0.92 | 0.16 | <0.001 | 12.58 | 0.60 | 0.02 | <0.001 | 12.20 | 0.56 | <0.001 | 0.69 | 0.05 | 0.04 | 0.158 |
| **Ethnicity (Other vs Chinese)** | -0.83 | 0.16 | <0.001 | 11.35 | 0.54 | 0.02 | <0.001 | 12.47 | 0.55 | <0.001 | -1.71 | -0.14 | 0.04 | <0.001 |
| **Marital status (Single vs Married)** | -0.81 | 0.23 | <0.001 | -2.66 | -0.13 | 0.04 | <0.001 | -1.13 | 0.82 | 0.168 | -1.98 | -0.16 | 0.06 | 0.005 |
| **Marital status (Divorced vs Married)** | 0.31 | 0.09 | 0.001 | 0.78 | 0.04 | 0.01 | 0.008 | 1.41 | 0.33 | <0.001 | -1.07 | -0.08 | 0.02 | <0.001 |
| **Working status (Employed vs Housewife)** | -0.32 | 0.08 | <0.001 | -1.91 | -0.09 | 0.01 | <0.001 | -2.45 | 0.27 | <0.001 | 0.78 | 0.06 | 0.02 | 0.001 |
| **Working status (Unemployed vs Housewife)** | -1.28 | 0.55 | 0.019 | -0.92 | -0.05 | 0.08 | 0.587 | -1.76 | 1.94 | 0.363 | 0.88 | 0.07 | 0.13 | 0.602 |
| **Working status (Retired vs Housewife)** | -0.42 | 0.18 | 0.017 | -1.31 | -0.06 | 0.03 | 0.016 | -2.97 | 0.62 | <0.001 | 2.46 | 0.19 | 0.04 | <0.001 |
| **Number of children (1 vs 0)** | -0.48 | 0.22 | 0.029 | -0.25 | -0.01 | 0.03 | 0.714 | 2.76 | 0.78 | <0.001 | -3.77 | -0.30 | 0.05 | <0.001 |
| **Number of children (2 vs 0)** | -0.35 | 0.19 | 0.069 | -1.02 | -0.05 | 0.03 | 0.080 | 3.70 | 0.67 | <0.001 | -6.07 | -0.50 | 0.05 | <0.001 |
| **Number of children (3+ vs 0)** | 0.15 | 0.18 | 0.392 | -1.63 | -0.08 | 0.03 | 0.003 | 6.91 | 0.62 | <0.001 | -10.87 | -0.92 | 0.04 | <0.001 |
| **Menopausal status (Post vs Pre)** | - | - | - | 1.90 | 0.09 | 0.02 | <0.001 | 7.37 | 0.42 | <0.001 | -6.73 | -0.55 | 0.03 | <0.001 |
| **Ever oral contraceptives (Yes vs No)** | -0.01 | 0.07 | 0.900 | - | - | - | - | - | - | - | -0.37 | -0.03 | 0.02 | 0.091 |
| **Ever HRT (Yes vs No)** | -0.17 | 0.10 | 0.090 | -0.42 | -0.02 | 0.02 | 0.174 | -3.26 | 0.35 | <0.001 | 4.04 | 0.31 | 0.02 | <0.001 |
| **Family history of breast cancer (Yes vs No)** | -0.14 | 0.21 | 0.515 | - | - | - | - | -1.99 | 0.74 | 0.007 | 3.38 | 0.26 | 0.05 | <0.001 |

**Supplementary Table 2**. Multivariable adjusted linear regression estimates of breast size and breast component measurements in a subset of women with children (*n*=21,073). Breast size and dense area were square-root transformed. * denotes back-transformed values for ease of interpretation.

|  | **Bust line (cm)** | | | **Breast area (cm^2^)** | | | | **Nondense area (cm^2^)** | | | **Dense area (cm^2^)** | | | |
| --- | --- | --- | --- | --- | --- | --- | --- | --- | --- | --- | --- | --- | --- | --- |
|  | **BETA** | **SE** | **P** | **BETA*** | **BETA** | **SE** | **P** | **BETA** | **SE** | **P** | **BETA*** | **BETA** | **SE** | **P** |
| **Age (55-59 vs <55)** | 0.16 | 0.09 | 0.063 | 1.86 | 0.09 | 0.01 | <0.001 | 4.70 | 0.32 | <0.001 | -3.59 | -0.31 | 0.02 | <0.001 |
| **Age (60+ vs <55)** | 0.21 | 0.09 | 0.023 | 3.69 | 0.18 | 0.02 | <0.001 | 9.91 | 0.35 | <0.001 | -8.22 | -0.74 | 0.02 | <0.001 |
| **BMI (30+ vs 25-29)** | 7.94 | 0.13 | <0.001 | 13.99 | 0.66 | 0.02 | <0.001 | 16.00 | 0.45 | <0.001 | -1.85 | -0.16 | 0.03 | <0.001 |
| **BMI (20-24 vs 25-29)** | -7.40 | 0.08 | <0.001 | -12.07 | -0.61 | 0.01 | <0.001 | -13.62 | 0.28 | <0.001 | 1.74 | 0.15 | 0.02 | <0.001 |
| **BMI (<20 vs 25-29)** | -16.16 | 0.12 | <0.001 | -25.17 | -1.31 | 0.02 | <0.001 | -29.96 | 0.44 | <0.001 | 6.06 | 0.49 | 0.03 | <0.001 |
| **Ethnicity (Malay vs Chinese)** | -0.64 | 0.15 | <0.001 | 11.1 | 0.53 | 0.02 | <0.001 | 13.68 | 0.55 | <0.001 | -3.67 | -0.32 | 0.04 | <0.001 |
| **Ethnicity (Indian vs Chinese)** | -1.16 | 0.17 | <0.001 | 12.25 | 0.58 | 0.03 | <0.001 | 11.14 | 0.58 | <0.001 | 1.63 | 0.14 | 0.04 | 0.001 |
| **Ethnicity (Other vs Chinese)** | -1.05 | 0.16 | <0.001 | 10.85 | 0.52 | 0.02 | <0.001 | 11.55 | 0.57 | <0.001 | -1.17 | -0.10 | 0.04 | 0.011 |
| **Marital status (Single vs Married)** | 1.04 | 1.07 | 0.331 | 0.28 | 0.01 | 0.16 | 0.933 | -0.44 | 3.77 | 0.908 | 0.67 | 0.06 | 0.26 | 0.827 |
| **Marital status (Divorced vs Married)** | 0.27 | 0.10 | 0.004 | 0.59 | 0.03 | 0.01 | 0.048 | 1.14 | 0.34 | 0.001 | -0.84 | -0.07 | 0.02 | 0.002 |
| **Working status (Employed vs Housewife)** | -0.23 | 0.08 | 0.004 | -1.58 | -0.08 | 0.01 | <0.001 | -1.97 | 0.28 | <0.001 | 0.52 | 0.04 | 0.02 | 0.020 |
| **Working status (Unemployed vs Housewife)** | -1.57 | 0.79 | 0.047 | -0.28 | -0.01 | 0.12 | 0.911 | -1.78 | 2.79 | 0.524 | 1.51 | 0.13 | 0.19 | 0.508 |
| **Working status (Retired vs Housewife)** | -0.37 | 0.2 | 0.059 | -0.85 | -0.04 | 0.03 | 0.165 | -2.32 | 0.69 | 0.001 | 1.98 | 0.17 | 0.05 | <0.001 |
| **Number of children (2 vs 1)** | 0.07 | 0.16 | 0.686 | -1.02 | -0.05 | 0.02 | 0.045 | 0.55 | 0.57 | 0.335 | -2.00 | -0.17 | 0.04 | <0.001 |
| **Number of children (3+ vs 1)** | 0.36 | 0.16 | 0.022 | -2.34 | -0.11 | 0.02 | <0.001 | 2.43 | 0.55 | <0.001 | -5.82 | -0.52 | 0.04 | <0.001 |
| **Age at first birth (22-26 vs <22)** | -0.30 | 0.08 | <0.001 | -1.34 | -0.07 | 0.01 | <0.001 | -2.63 | 0.30 | <0.001 | 1.81 | 0.15 | 0.02 | <0.001 |
| **Age at first birth (>=27 vs <22)** | -0.62 | 0.1 | <0.001 | -2.47 | -0.12 | 0.02 | <0.001 | -4.01 | 0.36 | <0.001 | 2.36 | 0.20 | 0.02 | <0.001 |
| **Breastfed (No vs Yes)** | -0.13 | 0.08 | 0.104 | -0.11 | -0.01 | 0.01 | 0.659 | -0.72 | 0.28 | 0.011 | 0.88 | 0.07 | 0.02 | <0.001 |
| **Menopausal status (Post vs Pre)** | - | - | - | 1.92 | 0.09 | 0.02 | <0.001 | 7.51 | 0.43 | <0.001 | -6.4 | -0.57 | 0.03 | <0.001 |
| **Ever oral contraceptives (Yes vs No)** | -0.03 | 0.07 | 0.705 | - | - | - | - | - | - | - | -0.44 | -0.04 | 0.02 | 0.031 |
| **Ever HRT (Yes vs No)** | -0.19 | 0.1 | 0.068 | -0.46 | -0.02 | 0.02 | 0.157 | -3.31 | 0.37 | <0.001 | 3.81 | 0.31 | 0.03 | <0.001 |
| **Family history of breast cancer (Yes vs No)** | -0.16 | 0.22 | 0.475 | - | - | - | - | -2.07 | 0.78 | 0.008 | 3.16 | 0.26 | 0.05 | <0.001 |
